# Peptide hormones and bile acids shaping immune tolerance of the liver: implications and applications

**DOI:** 10.1093/procel/pwaf096

**Published:** 2025-11-09

**Authors:** Gustav van Niekerk, Yana Kumpanenko, Joran Degryse, Johan Fevery, Kai Dallmeier

**Affiliations:** Laboratory of Molecular Vaccinology and Vaccine Discovery, Division of Virology, Antiviral Drug and Vaccine Research, Department of Microbiology, Immunology and Transplantation, Rega Institute, KU Leuven, Leuven 3000, Belgium; Laboratory of Molecular Vaccinology and Vaccine Discovery, Division of Virology, Antiviral Drug and Vaccine Research, Department of Microbiology, Immunology and Transplantation, Rega Institute, KU Leuven, Leuven 3000, Belgium; Laboratory of Molecular Vaccinology and Vaccine Discovery, Division of Virology, Antiviral Drug and Vaccine Research, Department of Microbiology, Immunology and Transplantation, Rega Institute, KU Leuven, Leuven 3000, Belgium; Hepatology Unit, Faculty of Medicine, KU Leuven, Leuven 3000, Belgium; Laboratory of Molecular Vaccinology and Vaccine Discovery, Division of Virology, Antiviral Drug and Vaccine Research, Department of Microbiology, Immunology and Transplantation, Rega Institute, KU Leuven, Leuven 3000, Belgium

**Keywords:** tolerance, immunoendocrinology, bile acids, GLP-1, xenografts, nutrition

## Abstract

Postprandially reabsorbed bile acids, along with various peptide hormones released following a meal, orchestrate complex events associated with digestion and prepare the body for the disposal of incoming nutrients by regulating metabolism. Interestingly, these factors have also been shown to modulate immune function. For example, recent interest in weight-loss agents such as semaglutide has demonstrated their ability to attenuate inflammation and provide benefits in diverse clinical contexts characterized by inflammatory responses. This raises an important question: why do hormones with well-established roles in digestion and metabolism also influence immunity? Here, we propose that the immune-regulatory activity of peptide hormones, together with postprandially reabsorbed bile acids, contributes to another remarkable phenomenon: the exceptional immune tolerance of the liver. While it is well established that the liver is an immunologically tolerant organ, the precise mechanisms underlying this skewed immunological tone remain poorly understood. Hepatic immune tolerance has generally been considered an intrinsic property of the liver, arising from autonomous mechanisms. Here, we highlight that various entero-pancreatic endocrine factors delivered to the liver via the portal vein activate cAMP signalling, thereby promoting immune tolerance and attenuating inflammatory tone within the liver. Critically, because these endocrine factors reach the liver at elevated concentrations through the portal vein before dilution in the systemic circulation, they profoundly shape the hepatic immune environment. Physiologically, this system ensures that the liver tolerates diet- and gut-derived inflammogens. Finally, we discuss several implications of this mechanism.

## Introduction

The exceptional immune tolerance of the liver is evident from a number of observations. One example is the profound tolerance observed following liver transplants, where, unlike for any other organ, a sizeable number of liver transplant recipients are eventually able to be weaned off immunosuppressive drugs (Londoño et al., 2013). This tolerance is also observed in other animals, such as pigs (Calne et al., 1969). In mice, the liver stood out as the only organ where the majority of allografts were not rejected, despite strain-specific MHC (major histocompatibility complex) mismatches between donor and recipient mice (Zhang et al., 1996). Additionally, the liver’s notable tolerance may render it more susceptible to pathogens. Indeed, the ‘liver provides a favourable environment for escaping immune responses’ (Protzer et al., 2012), with various pathogens demonstrating tropism for the liver (Horst et al., 2016). Similarly, the tolerogenic environment of the liver poses a significant challenge in treating metastatic cancer. Historically, the prognosis for patients with liver metastases originating from gastric cancer has been described as “dismal” (Kakeji et al., 2010). Unfortunately, progress has been limited, with surgical removal of respectable cancer being the only curative option in most cases (Uutela et al., 2023). A recent clinical trial (NCT02597348) reported a significant increase in survival for patients with colorectal liver metastases who received a liver transplant compared to standard chemotherapy alone (Adam et al., 2024). Liver metastasis also possess a challenge to onco-immunological strategies, such as immune checkpoint inhibitors. For example, it was recently noted that immune checkpoint inhibitors deliver poor results in the context of colorectal cancer following liver metastasis (Chen et al., 2023). Taken together, these observations underscore how immune tolerance within the liver compartment may complicate the clearance of pathogens and the effectiveness of cancer immune surveillance.

Understanding how hepatic tolerance manifests may thus pave the way for the development of strategies to ‘unveil’ this tolerogenic environment, facilitating more effective treatment of metastatic cancer and chronic liver infections. Conversely, insights into how the liver sustains such profound tolerance could lead to the development of novel anti-inflammatory strategies. Moreover, an interesting question arises: could the immune tolerance mechanisms at play in the liver be genetically engineered into xenografts? For example, despite 69 gene edits, a patient receiving a pig *kidney* survived for only 8 weeks. In contrast, a pig *liver* edited with six genes showed no signs of rejection for at least 130 days (implanted on 17 May, with no signs of organ rejection reported on 30 September 2024) (Brouillette, 2024).

The exact underlying mechanisms driving liver-specific tolerance remain to be fully elucidated. Here, we highlight the immune-modulatory effect of the endocrine response to food as a key driver of the striking immune tolerance observed in the liver. Several gastrointestinal peptide hormones released following a meal, along with postprandial reabsorbed bile acids (BAs) do not only regulate digestion and metabolism but also have been shown to exert immune-regulatory functions. The unique circulatory dynamics of the portal vein ensure that these endocrine factors reach the liver first and at elevated levels prior to be diluted in the systemic circulation, possibly shaping immunological tolerance particularly within the hepatic compartment. Finally, it should also be noted that various pharmacological agents such as semaglutide (marketed as Ozemic^®^/Wegovy^®^), tirzepatide (Mounjaro^®^), or retatrutide, currently used or investigated for weight loss, activate the same receptors as gastrointestinal peptide hormones do, suggesting that such weight loss agents also directly affect immune function.

## Why the liver needs to be tolerogenic

Following a meal, blood flow to the gastrointestinal tract increases, accompanied by a corresponding rise in blood flow through the hepatic portal vein. The portal vein transports harmless, diet-derived antigens as well as microbial components from the gut microbiota, which, if not efficiently cleared by the liver, could elicit severe inflammatory responses (Robinson et al., 2016). This phenomenon is exemplified by the systemic shock and inflammation occasionally observed following a transjugular intrahepatic portosystemic shunt (Jalan et al., 2011; Macnaughtan et al., 2017) or in animal models following portacaval shunting (García et al., 2011). In these scenarios, unfiltered blood from the hepatic portal vein is partially diverted directly into systemic circulation, resulting in systemic inflammation.

Another major source of inflammation includes diet-derived toxins, which can cause death of liver cells and lead to sterile inflammation. To discourage predation, plants have evolved a wide range of phyto-toxins, which, in turn, selected for liver detoxifying enzymes in herbivores, resulting in an evolutionary arms race between plants and herbivores (Steinberg, 2012). Domesticated plants have been selectively bred to produce less toxins, leading to relaxed evolutionary pressure. However, undomesticated plants can cause liver injury. Indeed, various plant-derived herbal or dietary supplements remain a significant source of liver damage (Navarro et al., 2017), highlighting the evolutionary pressure that plant-based foods may have exerted on the liver to tolerate and mitigate the hepatotoxic effects of these phyto-toxins. Similarly, various fungi are also toxic and cause profound liver damage. This is best exemplified by aflatoxins produced by moulds in the *Aspergillus* genus, which infect various food crops. Not only is aflatoxin a known hepatocarcinogen (Liu et al., 2012), but it is also implicated as a major contributing factor to childhood stunting in low-income countries in Africa (Rasheed et al., 2021). As a filter organ, the liver receives a high dose of these toxins via the portal vein, which in turn leads to cell death, necessitating the need to tolerate such sterile inflammation. This also explains the liver’s extraordinary regenerative capacity, as the attrition of cells resulting from dietary toxins must be replenished.

**Figure 1.**
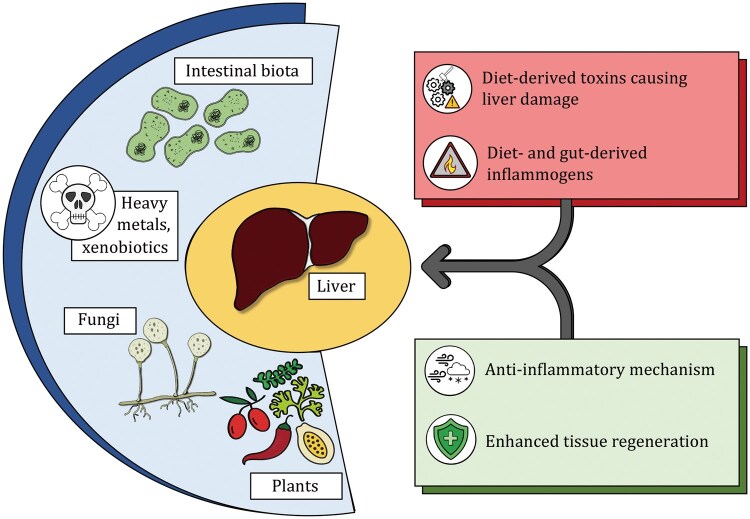
**The role the liver plays as a filtering organ has exerted significant evolutionary pressures, positioning it as a uniquely tolerogenic compartment**. First, diet-derived antigens and inflammogens from the intestinal microbiota must be processed without eliciting unnecessary inflammatory responses. Second, the liver must scavenge and manage toxins that inevitably cause tissue damage, necessitating potent coping mechanisms. These toxins may include plant- and fungal-derived compounds, as well as environmental contaminants such as heavy metals in water or xenobiotics generated during cooking and food preparation. To meet these challenges, the liver has evolved not only an unparalleled capacity for tissue regeneration but also a remarkable ability to tolerate sterile inflammation.

In summary, besides tolerating elevated levels of gut-derived inflammogens (e.g., lipopolysaccharides [LPS], plant lectins, mycotoxins in fungi), the liver must also detoxify diet-derived toxins (Fig. 1). As a filtering organ, the liver receives high concentrations of these noxious agents via the portal vein, which can occasionally induce cell death, necessitating tolerance to recurring inflammatory insults. This requirement for tolerance also explains the liver’s exceptional regenerative capacity, as the continuous attrition of cells due to dietary toxins must be compensated for by efficient cellular renewal.

## Postprandial reabsorbed bile acids

Using a combination of spatial transcriptomics and intravital imaging, it was recently confirmed that the liver is anatomically organized into zones with distinct immunological characteristics (Miyamoto et al., 2024). Notably, periportal vein zones were found to exhibit lower levels of inflammation, a phenomenon partially mediated by a subpopulation of tolerogenic Kupffer cells. These cells were characterized by the expression of Marco and increased production of anti-inflammatory cytokines, such as *Il1rn* and *Tgfb1*. These tolerogenic macrophages, located at the ‘gateway’ (periportal vein zones), appear to play a crucial role in maintaining tolerance to gut-derived pathogens. Genetic ablation of these cells resulted in increased liver inflammation, underscoring their importance in preserving hepatic immune homeostasis.

At first glance, these observations might have been interpreted as signifying a liver-intrinsic tolerance mechanism based on the distribution of tolerogenic immune cell populations within the liver. Crucially, however, the authors demonstrated that the presence of tolerogenic Marco^+^ cells is influenced by factors derived from the intestinal microbiota (Miyamoto et al., 2024): In mice raised in germ-free environments (i.e., devoid of intestinal microbiota), the population of Marco^+^ cells was significantly diminished. Further investigation identified isoallolithocholic acid, a secondary BA metabolite, as a key mediator in promoting Marco^+^ tolerogenic macrophages. These findings reveal that extrinsic factors, such as ­microbiota-derived secondary BAs metabolites, can substantially contribute to the establishment of liver immune tolerance.

Following a meal, the majority of BAs and their secondary metabolites, derived from bacterial metabolism, are reabsorbed and transported back to the liver via the portal vein, where they are recycled into the bile pool. However, these BAs, along with their secondary metabolites produced by bacterial metabolism, act as endocrine factors influencing a wide range of physiological systems. Notably, BAs are well-established as immune-regulatory agents (Fiorucci et al., 2018, 2022, 2024, 2025; Fuchs and Trauner, 2022; Sipka and Bruckner, 2014), delivering a potent anti-inflammatory signal to the liver. This influx of anti-inflammatory BAs not only shapes liver physiology, such as the differentiation of Marco^+^ Kupffer cells, but also modulates the activity of peripheral immune cells infiltrating the liver.

In the context of immune regulation, the Takeda G protein-coupled receptor 5 (TGR5), a transmembrane BA receptor, and the farnesoid X receptor (FXR), a nuclear receptor, have been extensively studied. Culturing human dendritic cells (DCs) derived from peripheral blood monocytes in the presence of BAs resulted in stunted expression of pro-inflammatory cytokines such as IL-12 and TNF (Ichikawa et al., 2012). Similarly, in an experimental autoimmune uveitis model, BAs promoted DC tolerance via the TGR5-cAMP-PKA pathway, suppressing NF-κB activation and thereby attenuating immune activation (Hu et al., 2021). These findings suggest that BAs play a pivotal role in modulating how DCs present antigens to immune cells.

As mentioned, the role of BAs in regulating immune responses has been extensively reviewed (Fiorucci et al., 2018, 2022, 2024, 2025; Fuchs and Trauner, 2022; Sipka and Bruckner, 2014). While the majority of studies support an anti-inflammatory function for BAs, notable exceptions exist. For instance, in a mouse model of graft-versus-host disease, pharmacological activation of FXR increased mortality, whereas genetic deletion of the FXR receptor in donor T cells improved survival outcomes (Lindner et al., 2024). BAs may also promote inflammation indirectly. In a mouse model of cholestatic liver injury, FXR activation was shown to increase the expression of P-selectin, which in turn facilitated neutrophil retention and exacerbated liver injury (Zhang et al., 2025).

Several factors likely contribute to the conflicting findings regarding the immunological effects of BAs. One important consideration is that BAs can activate a range of receptors beyond FXR and TGR5, including pregnane X receptor (PXR), the vitamin D receptor, and retinoic acid receptor-related orphan receptor γ (RORγ), among others. This complexity is further compounded by the fact that BA signalling outcomes are highly dependent on cell-specific signalling contexts. For example, while RORγ is generally associated with pro-inflammatory responses in T helper 17 (Th17) cells (Jetten et al., 2018), it has also been shown that RORγt (also known as RORγ2) plays a critical role in maintaining colonic Treg cells, with BAs such as lithocholic acid and 3-oxo-lithocholic acid acting as ligands for this nuclear receptor (Song et al., 2020). The mechanistic basis for these seemingly contradictory roles of RORγt was recently elucidated: phosphorylation of serine 182 on RORγt was found to suppress Th17 hyperactivation while simultaneously promoting IL-10 production by RORγt^+^ Treg cells (Ma et al., 2022). These findings suggest that receptor signalling can be substantially modulated by crosstalk with kinase signalling pathways.

In addition to the diversity of BA receptors, differential expression of these receptors across immune cell types further contributes to the heterogeneity of BA-mediated immune responses. For example, compared to bone marrow-derived macrophages, Kupffer cells exhibit higher levels of FXR expression and display a stronger anti-inflammatory response to FXR agonist pre-treatment, as evidenced by reduced secretion of pro-inflammatory cytokines such as IL-6, IL-1β, and TNF in a mouse model of ischemia-reperfusion liver injury (Jin et al., 2020). Likewise, expression levels of FXR and TGR5 are significantly lower in T and B lymphocytes compared to other immune cell populations (Fiorucci et al., 2018). These differences in receptor expression suggest that BAs may differentially modulate distinct compartments of the immune system depending on the specific cell types involved.

Another consideration is that the composition of the BA pool is influenced by the microbiome, as bacterial species vary in their metabolism of BAs (Collins et al., 2023). In turn, BAs differ in their affinity for and effects on these receptors. For instance, while chenodeoxycholic acid is a potent FXR agonist, other BAs such as ursodeoxycholic acid can act as FXR antagonists (Mueller et al., 2015). Similarly, while primary BAs have been shown to upregulate CXCL16 expression on liver sinusoidal endothelial cells (LSECs), secondary BAs, produced through microbial metabolism of primary BAs, tend to suppress CXCL16 expression. This, in turn, regulates the trafficking of CXCR6^+^ natural killer T (NKT) cells, which are recruited to the liver in response to CXCL16 (Ma et al., 2018). These findings highlight a potentially important mechanism by which the gut microbiota may modulate systemic inflammatory tone through its influence on the composition of the BA pool.

Inflammatory stimuli can also modulate the expression of BA receptors. For example, inflammation has been shown to reduce the expression of FXR and PXR in liver-infiltrating immune cells in mice with dextran sodium sulphate-induced colitis (Negroni et al., 2020). Similarly, FXR expression is suppressed by NF-κB activation (Gadaleta et al., 2011). Adding to this complexity, the inflammatory milieu can also alter both hepatic BA synthesis and the composition of the intestinal microbiota, thereby changing the overall BA profile (Lindner et al., 2024). Notably, this implies that the effects of BAs may vary when assessed in the setting of pre-existing inflammation versus when administered as a preventive intervention prior to an inflammatory challenge.

BAs also exert effects beyond receptor-mediated signalling. The physicochemical properties of bile salts, particularly their amphipathic nature, are essential for the emulsification of dietary fat droplets. However, in the case of hydrophobic BAs, cellular membrane function, including that of organelles such as mitochondria, can be severely disrupted (Schölmerich et al., 1984; Schulz et al., 2013). Consequently, chronic exposure to elevated BA levels may induce cellular stress and even necrotic cell death, thereby promoting a pro-inflammatory environment that may obscure the immunomodulatory effects mediated by BA receptors. This phenomenon may be especially relevant in pathological conditions such as cholestasis, where impaired bile flow leads to BA spillover and consequent tissue damage. In such contexts, liver injury may create an inflammatory milieu that does not accurately reflect the effects of BA receptor signalling. These considerations are also critical when interpreting studies that employ supraphysiological BA doses or prolonged exposure.

Taken together, while BAs are generally associated with anti-inflammatory effects, several factors may account for conflicting findings in the literature. These include the specific BA metabolites involved, the pre-existing inflammatory context, the composition of the immune cell population, and crosstalk between various signalling pathways. Moreover, the observation that certain BAs, particularly those with hydrophobic properties, can disrupt membrane integrity and induce cellular stress or even necrotic cell death suggests an additional mechanism by which BAs may promote inflammation. In this scenario, the physicochemical properties of BAs could indirectly trigger immune responses through the release of damage-associated molecular patterns (DAMPs) from necrotic cells or via cytokine release as a consequence of cellular stress.

## Gastric peptide hormones as immune modulators

Feeding triggers a comprehensive endocrine response that not only regulates the complex processes of digestion but also alters metabolism to manage the bulk disposal of incoming nutrients. A variety of peptide hormones, released by the gastrointestinal tract and pancreas, play a key role in orchestrating these processes. Strikingly, these peptide hormones have also been shown to influence the immune system.

Following a meal, cholecystokinin (CCK) is secreted by enteroendocrine I cells in the small intestine. CCK performs several critical regulatory functions in digestion, including promoting satiety, reducing gastric motility, and stimulating bile release from the gallbladder. Similarly, gastrin, another closely related peptide hormone, is secreted by the stomach and duodenum after a meal. Gastrin induces the secretion of hydrochloric acid by parietal cells in the stomach, facilitating digestion. Importantly, both CCK and gastrin have been found to regulate various aspects of immune function, highlighting their dual roles in digestion and immune modulation.

We have recently reviewed studies spanning several decades that report immune-modulatory roles for CCK and gastrin (van Niekerk et al., 2024). For instance, CCK may contribute to a tolerogenic environment by promoting Treg differentiation (Zhang et al., 2014) or by stunting IL-1β production by macrophages following LPS challenge (Li et al., 2007). Similarly, gastrin has been shown to increase intracellular cAMP levels in macrophages, leading to attenuation of immune functions such as chemotaxis and phagocytosis (De La Fuente et al., 1996). This observation is unexpected, as the gastrin receptor (CCK2R) typically signals through calcium-dependent pathways rather than cAMP-mediated mechanisms. However, other studies suggest that gastrin can regulate leukocyte–endothelial cell interactions, enhancing leukocyte rolling and adhesion, which exacerbates inflammation during *Helicobacter pylori* infection (Álvarez et al., 2006). Thus, while CCK appears to have predominantly anti-inflammatory effects, the role of gastrin in immune modulation remains less clear and context-dependent.

Another gastric peptide hormone with demonstrated immune-modulating functions is glucagon-like peptide-1 (GLP-1), with numerous studies demonstrating that GLP-1 receptor agonists (GLP-1RAs) exert anti-inflammatory effects (Drucker, 2024). As we recently highlighted, the widespread use of these weight-loss agents may have broader implications, including potential effects on vaccine responses (van Niekerk et al., 2024). Semaglutide, the active ingredient in Ozempic and Wegovy, was initially developed for the treatment of type 2 diabetes, is also having a transformative impact on weight loss and the management of obesity-related pathologies. Its therapeutic potential in diabetes management arises from GLP-1’s ability to suppress glucagon release, thereby reducing gluconeogenesis, while simultaneously enhancing glucose-dependent insulin secretion. The appetite-suppressing properties of GLP-1RAs have garnered significant attention, particularly for their role in obesity management. As the use of these and other similar agents becomes more prevalent, an urgent need arises to elucidate the impact of this class of medications on immune function.

Glucose-dependent insulinotropic polypeptide (GIP) is released by K cells in the duodenum following a meal. Like GLP-1, GIP also acts as an incretin, stimulating insulin release, and is being explored as a therapeutic target for diabetes and weight loss. Interestingly, the GIP receptor (GIPR) is expressed on T cells, myeloid cells, and their precursors (Pujadas et al., 2020), and has been found to regulate the inflammatory response. On the one hand, GIP release is stimulated in mice following an IL-1β challenge, and treatment with a GIPR antagonist resulted in reduced IL-6 and TNF release after an LPS challenge (Kahles et al., 2016), indicating that GIP may have pro-inflammatory effects. In contrast, other studies highlight GIP’s role in attenuating 5-fluorouracil-induced gut inflammation (Hammoud et al., 2024). Similarly, GIP has been implicated in restraining inflammation by suppressing the expression of pro-inflammatory mediator S100A8 (Mantelmacher et al., 2019). Here, it was observed that adipose tissue macrophages deficient in GIPR exhibited increased S100A8 expression, whereas GIP effectively downregulated S100A8 expression in GIPR-competent immune cells. Considering the diverse roles of the S100 calcium-binding protein heterodimer S100A8/A9 in upregulating an inflammatory tone (Wang et al., 2018), it is plausible that GIP exerts a significant impact on immune function through this pathway. These seemingly conflicting roles of GIP in pro- and anti-inflammatory responses suggest that its function is not yet fully elucidated, while also highlighting the immune-regulatory properties of this incretin hormone.

During fasting, low glucose levels stimulate pancreatic α cells to release glucagon, which promotes gluconeogenesis in the liver. However, glucagon is also transiently secreted following a meal, particularly after protein-rich intake (Ichikawa et al., 2023). This postprandial release likely reflects glucagon’s role in stimulating hepatic amino acid uptake, thereby facilitating postprandial amino acid disposal. Another function of postprandially released glucagon is to stimulate insulin secretion by pancreatic β cells (Svendsen et al., 2018). Thus, although glucagon levels are primarily elevated in the fasted state, a postprandial response is also a characteristic feature of its secretion.

It has long been known that various lymphoid cell lines express glucagon receptors and that glucagon signalling is functional in these cells, as evidenced by increases in intracellular cAMP (Koh et al., 1996). In sensitized mice challenged with an ovalbumin model of asthma, glucagon treatment alleviated airway hyperreactivity and the associated inflammatory response (Insuela et al., 2019). Similarly, glucagon has been shown to increase susceptibility to sepsis by attenuating both neutrophil chemotaxis and reactive oxygen species production (Insuela et al., 2021). Given that glucagon levels are often elevated in diabetic patients, the authors of the study suggested that this increase may contribute to the heightened susceptibility of diabetic individuals to various infections.

In contrast to these anti-inflammatory effects, glucagon has also been reported to increase the expression of IL-1β, IL-6, complement component 3, and C-reactive protein, as well as to enhance activation of the NLRP3 inflammasome in HepG2 cells (Andreozzi et al., 2023). It should be noted, however, that HepG2 is a hepatocellular carcinoma cell line, and that *in vitro* conditions may differ substantially from *in vivo* physiology. For example, depending on the metabolic state, cells maintained in culture flasks may experience hypoxic conditions, whereas even a simple washing step can expose them to partial oxygen pressures far exceeding those found in physiological tissue (Tan et al., 2024). These observations suggest that cellular stress (e.g., oxidative or hypoxic) may modulate the effects of glucagon.

Vasoactive intestinal peptide (VIP) is synthesized and released by gut-associated neurons in the gastrointestinal tract following a meal, subsequently reaching the liver at elevated levels through the hepatic portal vein. The immune-modulating effects of VIP have long been recognized (Delgado et al., 2002). Current evidence suggests that while VIP predominantly exerts broad anti-inflammatory functions, it also plays roles in promoting specific immune responses (Pozo et al., 2000). For instance, VIP has been shown to be crucial for protection against *Citrobacter rodentium* infection (Yu et al., 2021). Studies using *Vip*^−/−^ mice demonstrated that these mice were more susceptible to *C. rodentium* infection, exhibiting a blunted IL-22 response. Mechanistically, VIP was found to play a critical role in the recruitment and retention of group 3 innate lymphoid cells in the gastrointestinal tract. Notably, VIP was also shown to be essential for the maintenance of retinoic acid (RA)-producing DCs (Yu et al., 2021). This finding is particularly significant, as RA-producing DCs are often associated with pro-tolerogenic roles in both the gut (Cassani et al., 2012) and liver (Bhatt et al., 2014). Thus, VIP may contribute to mitigating tissue damage by modulating inflammation and promoting immune tolerance.

Other peptide hormones entering the portal vein exhibit less well-defined immune-regulatory functions. Early studies reported that lymphocytes express the secretin receptor (Rindi et al., 2004), but its potential immune-regulatory effects remain largely unexplored. Amylin, a hormone co-released with insulin by pancreatic β cells, also reaches the liver via the hepatic portal vein. Notably, amylin has been shown to promote the differentiation of Treg cells and the expression of TGF-β (Zhang et al., 2018), suggesting it may play a role in the immunological tolerance observed in the liver. These findings implicate amylin as another peptide hormone contributing to the unique tolerogenic environment of the liver.

**Figure 2.**
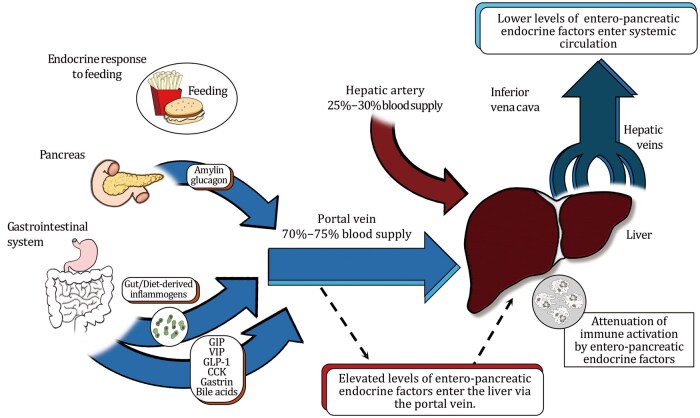
**Following a meal, gut-derived inflammogens originating from intestinal microbiota and food can activate immune responses within the liver compartment**. The immune-regulatory functions of various peptide hormones and BAs serve to synchronize the digestive process with anti-inflammatory function: as gut-derived inflammogens enter the portal vein, an influx of tolerogenic-­promoting immune modulation endocrine factors also enters, preventing severe inflammatory responses. As the majority of blood entering the liver (approximately 70%–75%) flows through the portal vein, which carries entero-pancreatic endocrine factors, the liver is expected to be profoundly influenced by their elevated levels. Following filtration through the liver, blood enters the systemic circulation, where the remaining endocrine factors are diluted. Prolonged cycles of exposure to these endocrine factors, released into the splanchnic circulation, may ultimately lead to histological adaptation in the liver (e.g., the accumulation of macrophages and Kupffer cells within the periportal vein zones).

At first glance, it may seem counterintuitive for hormones that regulate digestion and metabolic reprogramming to also influence immune function. However, following a meal, the significant increase in blood flow to the gastrointestinal tract, and the subsequent drainage into the portal vein, subjects the liver to inflammatory insults arising from gut- and diet-derived inflammogens, as well as potential tissue damage caused by ingested toxins. Assigning an immune-regulatory function to these hormones represents a mechanism for ensuring tolerance to inbound antigens (Fig. 2).

## Entero-pancreatic endocrine factors signalling via cAMP

As discussed, the unique vascular architecture of the hepatic portal vein plays a central role in establishing the liver’s distinctive tolerogenic environment. Reabsorbed BAs, along with entero-pancreatic peptide hormones, enter the liver via the portal vein. Because these endocrine factors reach the liver prior to dilution in the systemic circulation, their concentrated influx is likely to exert a pronounced influence on shaping immune tolerance within the hepatic compartment. Although these factors have been shown to modulate immune function, the mechanistic basis by which they exert these effects in immune cells remains poorly understood. Nevertheless, the downstream signalling pathways activated by these endocrine factors have been extensively characterized in other physiological systems and, as discussed below, reveal striking immunomodulatory mechanisms.

The majority of endocrine factors entering the liver via the portal vein are G protein-coupled receptors (GPCRs) that utilize cyclic AMP (cAMP) as a second messenger (i.e., they are Gs-coupled). This group includes GIP and GLP-1 (Mayendraraj et al., 2022), glucagon (Janah et al., 2019), VIP (Piper et al., 2022), and amylin (Cao et al., 2022), all of which induce intracellular signalling cascades by elevating cAMP levels. Similarly, the BA receptor TGR5 is also a GPCR that signals via cAMP (Chen et al., 2020). In contrast, gastrin exclusively activates CCK2R, which is coupled to Gq and signals primarily through intracellular calcium. CCK, however, activates both CCK1R (which elevates cAMP) and CCK2R (Zeng et al., 2020). The observation that the vast majority of these entero-pancreatic endocrine factors utilize cAMP as a second messenger implies that the hepatic compartment is exposed to an elevated cAMP tone. This is particularly significant given the well-established role of cAMP in suppressing inflammatory responses (Raker et al., 2016; Tavares et al., 2020).

**Figure 3.**
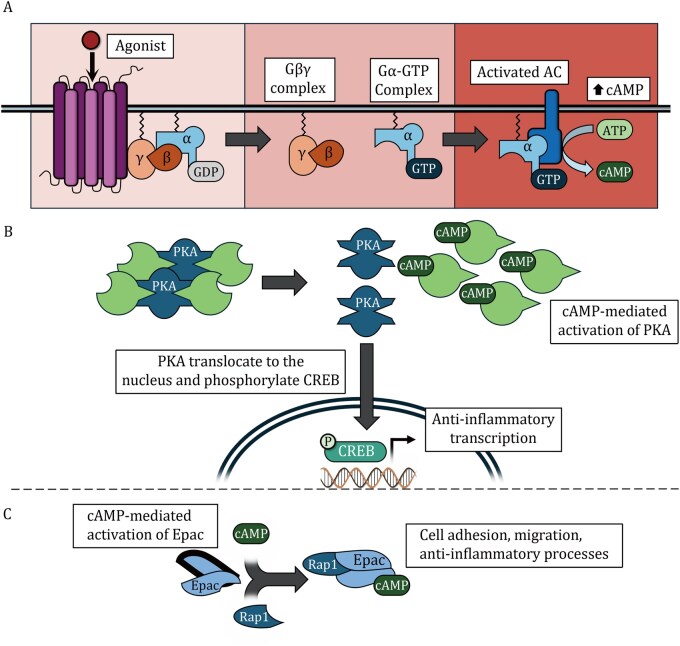
**Various entero-pancreatic endocrine factors signal via G protein-coupled receptors (GPCRs) that increase cAMP levels as a secondary messenger**. (A) Binding of agonist to extracellular receptor result in the conformational change in the receptor that permits the exchange of GDP for GTP. Once GTP is bound, Gα dissociates from the Gβγ subunits and is free to bind to and activate adenylyl cyclase (AC), an enzyme embedded in the plasma membrane. AC catalyses the conversion of ATP to cyclic AMP (cAMP). (B) In its inactive form, protein kinase A (PKA) exists as a tetramer composed of two regulatory subunits complexed with two catalytic subunits. An increase in intracellular cAMP levels leads to the binding of cAMP to the regulatory subunits, inducing conformational changes that cause dissociation of the regulatory and catalytic subunits. The liberated catalytic subunits are enzymatically active and phosphorylate serine/threonine residues on a wide range of target proteins. Upon translocation to the nucleus, PKA can phosphorylate the transcription factor CREB (cAMP response element-binding protein), thereby promoting the recruitment and assembly of transcriptional coactivators and facilitating the initiation of gene transcription. (C) Exchange protein directly activated by cAMP (Epac) signalling is also modulated by cAMP. Epac proteins (Epac1 and Epac2) function as guanine nucleotide exchange factors (GEFs), facilitating the exchange of GDP for GTP on the small GTPases Rap1 and Rap2, members of the Ras family. In their inactive state, Epac proteins adopt an autoinhibited conformation in which the regulatory domain occludes the catalytic GEF domain. Upon elevation of intracellular cAMP levels, cAMP binds to Epac, triggering a conformational change that relieves this autoinhibition. The exposed GEF domain then promotes the activation of Rap1 or Rap2 by catalysing the exchange of GDP for GTP.

Gs-coupled protein receptors increase intracellular cAMP levels by activating adenylyl cyclase (AC), which catalyses the conversion of ATP to cAMP (Fig. 3). This process is initiated by the binding of an agonist to the receptor, resulting in a conformational change that facilitates the exchange of GDP for GTP on the Gα subunit. This exchange triggers the dissociation of the Gα and Gβγ subunits. The liberated Gα subunit subsequently activates AC, leading to the production of cAMP from ATP. Among the best-characterized effectors of cAMP are protein kinase A (PKA) and the exchange protein directly activated by cAMP (Epac). Both have been implicated in the regulation of pathways involved in immune attenuation, resolution of inflammation, and the establishment of immune tolerance. PKA mediates its effects through several mechanisms, most notably via the phosphorylation and activation of the transcription factor CREB. In contrast, Epac activates Rap GTPases, thereby initiating distinct downstream signalling cascades that also contribute to immune regulation.

Many of the most prominent effects of PKA are mediated through its ability to phosphorylate and thereby activate the transcription factor CREB (Fig. 3B). One key anti-inflammatory mechanism attributed to CREB involves its antagonism of NF-κB-dependent transcription. For example, CREB can competitively bind to CREB-binding protein (CBP), thereby displacing the p65 subunit of NF-κB, which suggests a general feedback mechanism to prevent excessive inflammation (Parry and Mackman, 1997). In addition, CREB promotes the transcription of the inducible cAMP early repressor (ICER) by binding to cAMP response elements (CRE) in its promoter. ICER is primarily known as a negative feedback regulator that inhibits cAMP-dependent gene transcription. However, ICER has also been shown to inhibit NF-κB activity through direct interaction with the p65 subunit (Lv et al., 2017). Thus, CREB may antagonize NF-κB activity both directly, via competition for CBP, and indirectly, through ICER-mediated inhibition. Moreover, CREB regulates the expression of several anti-inflammatory genes. For instance, following TLR2 and TLR4 activation, phosphorylated CREB is recruited to the IL-10 promoter, thereby promoting IL-10 transcription and limiting excessive inflammatory responses (Sanin et al., 2015).

Another tolerance-promoting mechanism involving the cAMP–PKA–CREB axis is the regulation of non-canonical human leukocyte antigen (HLA)-G expression. While all nucleated cells express classical HLA class I molecules, the expression of HLA-G under steady-state conditions is largely restricted to compartments where immune activation must be tightly controlled, such as the placenta and immune-privileged sites like the cornea and thymus. HLA-G plays a critical role in promoting immune tolerance (Arnaiz-Villena et al., 2021), with its function most clearly demonstrated in mediating maternal–fetal tolerance during pregnancy (Ferreira et al., 2017). Furthermore, HLA-G has been implicated in immune evasion during viral infections (Jasinski-Bergner et al., 2022) and cancer (Lin and Yan, 2021). In this regard, cAMP has been shown to regulate HLA-G expression via CREB1 (Gobin et al., 2002), and dysregulated CREB-mediated transcription has been linked to increased HLA-G expression and immune evasion in cancer (Friedrich et al., 2020). Notably, HLA-G expression in the liver has been observed in pathological states (Amiot et al., 2015) and may contribute to immune evasion during chronic liver infections. However, its potential role in maintaining hepatic immune tolerance under steady-state conditions remains to be elucidated.

Epac modulates a variety of cellular responses, including those involved in anti-inflammatory pathways (Fig. 3C). For example, cAMP-mediated activation of Epac following adenosine A2A receptor stimulation has been shown to attenuate matrix-induced inflammation (Scheibner et al., 2009). Additionally, Epac contributes to the suppression of asthmatic airway inflammation (Chen et al., 2019) and provides protection against LPS-induced lung injury (Wang et al., 2018). Furthermore, Epac1 plays a key role in Treg function by antagonizing STAT3, a critical transcription factor downstream of T-cell receptor activation (Almahariq et al., 2015). There is also emerging evidence that Epac plays a role in host responses to respiratory viruses. For instance, silencing or pharmacological inhibition of Epac1 has been shown to reduce MERS-CoV replication (Tao et al., 2014), suggesting that the immune-attenuating effects of Epac1 may impair cell-autonomous defence mechanisms against viral infection. Taken together, these findings highlight the broad immunomodulatory role of Epac, particularly in limiting inflammation and contributing to immune tolerance.

Emerging evidence also implicates a role for Epac in immune regulation via modulation of IL-6 signalling. IL-6 can signal through two primary pathways: classic signalling, in which IL-6 binds to the membrane-bound IL-6 receptor (IL-6R) in complex with gp130, and trans-signalling, in which IL-6 binds to a soluble form of IL-6R (sIL-6R), enabling activation of gp130 on cells that do not express membrane-bound IL-6R. These two modes of IL-6 signalling are functionally distinct, with trans-signalling more commonly associated with pro-inflammatory responses and thus representing an attractive therapeutic target (Rose-John et al., 2023). Notably, in vascular endothelial cells, Epac has been shown to attenuate IL-6 trans-signalling by promoting the activation of suppressor of cytokine signalling 3 (SOCS3), a known negative regulator of cytokine signalling (Sands et al., 2006). This Epac-SOCS3 axis has also been implicated in the attenuation of IL-6-mediated pro-inflammatory signalling in other cell types (Huang et al., 2022). Therefore, cAMP, through Epac activation, likely plays a key role in dampening IL-6 trans-signalling as a mechanism to limit inflammation.

Evidence indicates that a similar cAMP/Epac-mediated mechanism might modulate IL-6 trans-signalling in immune cells. In this regard, recent findings indicate that classic IL-6 signalling exerts anti-inflammatory effects in T cells by promoting oxidative phosphorylation, whereas IL-6 trans-signalling favours a pro-inflammatory state by enhancing aerobic glycolysis (Xu et al., 2024). Notably, IL-6 trans-signalling biases T-cell differentiation towards Th17 cells, while classic IL-6 signalling promotes Treg differentiation. This observation is particularly intriguing, as it suggests that cAMP/Epac-mediated regulation of IL-6 signalling modes may influence the immunometabolic programming of immune cells.

cAMP has also been shown to play a comprehensive role in the development and function of Treg cells. Upon activation, Tregs upregulate intracellular cAMP levels, and the sustained elevation of cAMP is essential for their suppressive capacity (Klein et al., 2012). Beyond its role in Treg development, cAMP is also crucial for Treg-mediated immunoregulation. For example, it was recently demonstrated that in induced Treg (iTreg) cells, cAMP regulates the expression of CD39, a membrane-bound ecto-enzyme that hydrolyses extracellular ATP and ADP into AMP (Su et al., 2019). This mechanism is critical for controlling inflammation, as both ATP and ADP act as pro-inflammatory DAMPs. In the context of liver injury, such as that induced by diet-derived toxins, necrotic hepatocyte death can result in the release of extracellular ATP and ADP, thereby triggering inflammation. As such, the degradation of these nucleotides by CD39 may represent an important mechanism for preventing inflammation. Finally, Tregs also regulate immune responses in other cells by increasing their intracellular cAMP levels. For instance, in both B cells and CD4^+^ T cells, Treg-mediated elevation of cAMP suppresses the activity of NFATc1, a transcription factor involved in immune cell activation (Vaeth et al., 2011).

Given the diverse array of anti-inflammatory mechanisms mediated by cAMP, such as those involving Epac and the PKA–CREB axis, it is not surprising that pharmacological agents targeting cAMP signalling are also known to exert immunomodulatory effects. For instance, forskolin, which directly activates AC, and thereby increases intracellular cAMP levels, has been shown to exert anti-­inflammatory effects in various settings (Alasbahi and Melzig, 2012; Chen et al., 2019). Phosphodiesterases (PDEs) act as negative regulators of cAMP signalling by degrading cAMP into AMP. It is therefore expected that inhibiting PDE activity would enhance cAMP signalling, thereby promoting an immunosuppressive effect. Indeed, apremilast, a selective PDE4 inhibitor, is used clinically to treat autoimmune conditions such as psoriasis and psoriatic arthritis (Perez-Aso et al., 2015).

Further supporting the role of cAMP in controlling inflammation, several receptor agonists that elevate cAMP also promote immunosuppressive effects. Prostaglandin E2 (PGE2) receptor activation increases cAMP levels via Gs-coupled receptors and are known to modulate immune responses (Brudvik and Taskén, 2012). Similarly, β2-adrenergic receptor agonists, commonly used in the treatment of asthma, also possess well-documented immunosuppressive properties. Examples include salbutamol and terbutaline that increase cAMP levels by β2-adrenergic receptor signalling (Keränen et al., 2016). Additionally, adenosine A2A receptors, which are also Gs-coupled, are emerging as promising targets for immunomodulation. Indeed, A2A adenosine receptor agonists such as regadenoson are currently being explored in the context of organ transplantation (Lau et al., 2020).

cAMP also appears to play a key role in regulating inflammasome activity. In macrophages, the non-canonical caspase-11 inflammasome, typically involved in pyroptosis and the release of pro-inflammatory cytokines, is inhibited by cAMP. Specifically, elevated cAMP levels prevent activation of the caspase-11 inflammasome in response to cytosolic LPS through PKA activation (Chen et al., 2019). In the liver, scavenger receptors play a critical role in the uptake and clearance of LPS from circulation (Cabral et al., 2021; Guo et al., 2014). Given that the liver is constantly exposed to high levels of gut-derived LPS, the ability of cAMP–PKA signalling to suppress LPS-induced inflammasome activation may represent an essential tolerogenic mechanism. Furthermore, entero-pancreatic hormones that elevate intracellular cAMP levels may contribute to this axis of hepatic immune tolerance. This appears to be the case for BAs, as canonical NLRP3 inflammasome activity has also been shown to be negatively regulated by BAs via the TGR5-cAMP-PKA signalling pathway (Guo et al., 2016).

## cAMP-mediated immune tolerance in the liver

The preceding discussion highlights numerous observations implicating cAMP signalling in attenuating inflammatory responses and promoting immune tolerance. Notably, several studies have identified cAMP signalling as a key regulator in shaping hepatic immune tolerance. As noted earlier, isoallolithocholic acid was shown to promote the development of a tolerogenic Marco^+^ phenotype in Kupffer cells located in the periportal zone (Miyamoto et al., 2024). Notably, isoallolithocholic acid, which is a known agonist of TGR5 (Sun et al., 2023), and as mentioned, TGR5 is a GPCR that signals via the cAMP pathway (Chen et al., 2020). Thus, although the direct involvement of cAMP in driving the development of tolerogenic Marco^+^ Kupffer cells was not explicitly investigated in that study, it is likely that is oallolithocholic acid induced cAMP production via TGR5 activation, thereby contributing to the establishment of a tolerogenic phenotype in liver-resident immune cells.

Typically, exogenously derived epitopes are presented by phagocytic cells (e.g., macrophages or DCs) via MHC class II molecules. However, exogenous antigens can also be presented on MHC class I molecules through a process known as cross-presentation. In this context, LSECs are exceptionally efficient at presenting exogenous epitopes on MHC I to CD8^+^ T cells via cross-presentation (Limmer et al., 2000). This suggests that LSECs may play a central role in promoting tolerance to exogenous antigens encountered within the liver. Supporting this notion, recent findings have demonstrated that LSECs modulate CD8^+^ T-cell responses via cAMP, which functions as an ‘immune rheostat’ (Bosch et al., 2024). Mechanistically, antigen-presenting LSECs were shown to induce tolerance in CD8^+^ T cells through the release of PGE2, which in turn elevated cAMP levels and promoted antigen-specific unresponsiveness (Bosch et al., 2024). As a consequence, CD8^+^ T cells became unresponsive towards hepatocytes expressing viral antigens, thereby impairing antiviral immunity.

There is considerable interest in the role of IL-6 in the liver, as hepatocytes are highly sensitive to this pleiotropic cytokine, which regulates a broad range of hepatic functions, including regeneration and metabolic control (Cheng et al., 2022; Naseem et al., 2018; Schmidt-Arras and Rose-John, 2016). However, conflicting findings and ongoing controversy persist regarding its precise role in liver physiology and pathology. Indeed, a recent meta-analysis examining IL-6 in liver function reported ‘contradicting results in association with organ dysfunction’ (Murtha-Lemekhova et al., 2021). This uncertainty may, at least in part, arise from the context-dependent effects of IL-6, with its signalling outcomes modulated by crosstalk with other pathways active under distinct physiological and pathological conditions. As mentioned earlier, cAMP via the Epac-SOCS3 axis has been shown to attenuate IL-6 trans-signalling (Sands et al., 2006) and likely exerts similar modulatory effects in other cell types (Huang et al., 2022). This observation suggests a potential role for cAMP-mediated modulation of IL-6 signalling in the liver.

The unique physiology of IL-6 in the liver is exemplified by the observation that contracting skeletal muscle serves as a major source of IL-6, producing up to a 100-fold increase in circulating levels (Kistner et al., 2022; Nash et al., 2023). This surge in IL-6 plays a key role in maintaining glucose homeostasis during exercise: IL-6 not only stimulates pancreatic glucagon release (Chen et al., 2023), essential for hepatic gluconeogenesis, but also enhances cAMP-dependent glycogenolysis in the liver (Biazi et al., 2023). Thus, muscle-derived IL-6 facilitates the mobilization of hepatic glycogen stores to meet the elevated glucose demands of physical exertion. However, this mechanism presents a regulatory challenge: the liver must remain responsive to IL-6 to support glucose output while simultaneously avoiding the pro-inflammatory consequences typically associated with IL-6 signalling. In this context, glucose counterregulatory responses during exercise involve reduced insulin secretion alongside elevated glucagon levels (McCarthy et al., 2022), suggesting that glucagon-induced increases in cAMP may drive Epac activation. Indeed, glucagon has been shown to enhance Epac activity in liver cells *in vitro* (Cyphert et al., 2014; Sinclair et al., 2008). As noted, Epac antagonizes IL-6 trans-­signalling and its associated pro-inflammatory effects (Sands et al., 2006). This provides a plausible mechanism whereby exercise-induced elevations in glucagon increase hepatic cAMP, enabling Epac to mitigate the pro-inflammatory effects of IL-6 in the liver.

As previously mentioned, cAMP plays a key role in Treg function. In this context, there is also evidence to suggest that this mechanism may be particularly important in the liver. Early studies in pigs have demonstrated that co-transplantation of the liver with other tissues significantly delays graft rejection (Calne et al., 1969). Similarly, liver co-transplantation has been associated with enhanced immune tolerance in kidney (Taner et al., 2016) and heart (Wong et al., 2016) transplants in humans. A plausible mechanism underlying this phenomenon is that the transplanted liver expresses foreign graft antigens, which are rendered tolerogenic through the induction of antigen-specific Tregs. These Tregs may subsequently migrate to the co-transplanted organ, where they promote immune tolerance and contribute to the protection of the secondary graft.

Support for this perspective is provided by observations that liver-targeted allergen immunotherapy can promote tolerance to respiratory allergens. Liver-resident immune cells, such as Kupffer cells and LSECs, express high levels of mannose receptors, enabling preferential accumulation of synthetically mannosylated antigens in the liver. By exploiting this property, it was recently demonstrated that mannosylation of allergen-derived antigens can induce immune tolerance in the respiratory tract (Emiliano Gómez Medellín et al., 2025). In this context, Tregs generated in the liver were shown to play a critical role in establishing allergen-specific tolerance, suggesting that the liver actively promotes T-cell differentiation towards a Treg phenotype (Emiliano Gómez Medellín et al., 2025). This observation not only highlights the pro-tolerogenic capacity of the hepatic compartment but also demonstrates its systemic immunological impact.

Finally, the liver is one of the most NK cell-enriched organs in the body, with 30%–50% of all intrahepatic lymphocytes being NK cells. These cells display striking heterogeneity, with both resident and infiltrating NK cell populations acquiring unique tissue-imprinted phenotypes (Rebuffet et al., 2024). In this regard, cAMP likely plays a key role in contributing to this heterogeneity and in shaping NK cell phenotype. It has long been noted that elevations in cAMP suppress NK cell cytotoxic function (Goto et al., 1983). More recently, in the context of developing chimeric antigen receptor (CAR) NK cells, it was shown that cAMP acts as a key immunological ‘brake’ by activating the cAMP-responsive element modulator (CREM) (Rafei et al., 2025). Interestingly, during early implantation (first trimester), the placenta—another structure characterized by immune tolerance owing to its foetal origin—also harbours an abundance of NK cells (Faas and de Vos, 2017). In this setting, cAMP signalling has been implicated in the ‘re-education’ of decidual NK cells during endometrial decidualization, a process in which the uterus undergoes structural and functional remodeling to support pregnancy. Specifically, rising cAMP levels in endometrial stromal cells are associated with the reprogramming of decidual NK cells into CD56^bright^ NKs, which display low cytotoxicity while retaining a robust capacity for cytokine secretion (Jin et al., 2021). By contrast, the role of cAMP in liver-resident NK cells is less well defined, although early studies (Sirianni et al., 1992) indicate that cAMP-inducing hormones such as VIP and glucagon can attenuate NK cell function.

Taken together, it is clear that cAMP not only has a well-documented role in attenuating inflammation but also likely plays a key role in promoting immune tolerance in the liver. Although various entero-pancreatic endocrine factors have been shown to suppress inflammation through receptor-mediated increases in cAMP, their precise role in shaping hepatic tolerance via modulation of cAMP tone remains to be fully elucidated.

## cAMP signalling specificity and complexity

As mentioned, there are occasional conflicting reports regarding the effects of various endocrine factors on immune function. These discrepancies may, in part, reflect the intrinsic complexity of cAMP signalling, which can simultaneously influence multiple cellular processes. For example, cAMP has been shown to reduce neutrophil retention by limiting their adhesion to bronchial epithelial cells (Bloemen et al., 1997). However, cAMP can also prevent apoptosis in neutrophils, thereby extending their lifespan (Martin et al., 2001). In one context, cAMP signalling may therefore appear anti-inflammatory by reducing neutrophil adhesion and promoting their clearance from tissue. In another context, however, it may be interpreted as pro-inflammatory, as it prolongs the presence of ­neutrophils. This complexity highlights the limitations of categorizing cAMP signalling as purely pro- or anti-inflammatory, and underscores the importance of context when interpreting its immunological effects.

The complexity of cAMP signalling is further underscored by the observation that, although numerous hormone receptors utilize cAMP as a secondary messenger, they do not produce identical downstream effects. For instance, though both glucagon and GLP-1 treatments lead to a comparable increase in intracellular cAMP levels, only glucagon enhances the expression of ICER, which subsequently suppresses the transcription of insulin-related genes (Hussain et al., 2000). Similarly, while both forskolin (an activator of AC) and PGE2 receptor activation suppress TNF production, only forskolin also reduces IL-10 secretion (Dahle et al., 2005). This suggests that the receptor through which cAMP is elevated can confer specificity to cellular outcomes. Furthermore, they illustrate that both pro-inflammatory (e.g., TNF) and anti-inflammatory (e.g., IL-10) mediators can be simultaneously yet differentially regulated by cAMP signalling, depending on the context and pathway involved.

These findings may also have important implications for how cAMP interacts with other signalling pathways. For example, in thyroid cells, thyroid-stimulating hormone increases intracellular cAMP, which has been shown to activate mTOR, particularly complex 1 (mTORC1) ­(Blancquaert et al., 2010). In contrast, elevated cAMP has been reported to inhibit both mTORC1 and mTORC2 in mouse embryonic fibroblasts (Xie et al., 2011). This highlights the context-specific effects of cAMP signalling. Moreover, the case of mTOR is particularly noteworthy, as mTOR plays a pivotal role in immune cell activation by promoting anabolic processes required for cell growth and proliferation (Powell et al., 2012). Indeed, several immunosuppressive drugs used to prevent organ rejection in transplant patients, such as sirolimus and everolimus, act as mTOR inhibitors. These observations underscore the notion that cAMP can exert diverse and sometimes opposing effects depending on its crosstalk with other major signalling pathways.

One key mechanism underlying the diverse, and sometimes contrasting effects of cAMP signalling lies in the pattern and duration of cAMP signalling events. For instance, sustained elevations in cAMP are required for the induction of Tregs (Klein et al., 2012), whereas a transient increase in cAMP is observed following T-cell activation (Conche et al., 2009; Kanda and Watanabe, 2001). In this context, PDEs likely play a crucial role in regulating the decline in cAMP levels necessary to prevent the immunosuppressive effects typically associated with prolonged cAMP signalling (Bielenberg et al., 2024). These observations highlight the importance of the temporal dynamics of cAMP signalling in shaping its downstream immunological outcomes.

Another major feature of cAMP signalling that gives rise to specific effects is the spatial confinement of cAMP-related signalling events. Three key mechanisms contribute to the localization of cAMP signalling (Bock et al., 2024; Lefkimmiatis and Zaccolo, 2014). First, the trafficking of activated membrane receptors to intracellular compartments leads to the local activation of AC, creating spatial gradients of cAMP. For example, in neurons, dopamine receptors are trafficked into AC-containing endosomes and directed to specific cellular compartments to elicit defined signalling outputs (Ripoll and von Zastrow, 2023). Second, the localization of PDEs, which degrade cAMP, creates local ‘exclusion zones’ where cAMP is rapidly metabolized and thus rendered ineffective. For instance, the spatial regulation of β-adrenergic-stimulated cAMP signalling is mediated by compartmentalized PDE2, resulting in ‘cAMP dead zones’ (Mongillo et al., 2006). Finally, key scaffolding proteins such as A-kinase anchoring proteins (AKAPs) play a crucial role in organizing local signalosomes that spatially coordinate cAMP signalling events. In this regard, pre-treating DCs with AKAP inhibitors produced diverse effects upon lipopolysaccharide LPS challenge, highlighting the importance of AKAP-mediated localization of PKA in antigen presentation (Schillace et al., 2009).

Although this discussion has primarily focused on cAMP-mediated signalling downstream of receptor activation, emerging evidence suggests that the Gβγ complex (see Fig. 3) can also translocate to various intracellular membrane compartments, where it regulates a range of additional signalling pathways (Kankanamge et al., 2022). As a result, the downstream effects of GPCR activation may include Gβγ-mediated processes that extend beyond canonical cAMP signalling. This may elucidate certain observations, such as the modulation of cytokine release in CD4^+^ T cells by a VIP analogue through both cAMP-­dependent and cAMP-independent mechanisms (Wang et al., 1999).

Finally, another major feature of GPCRs is that, although various agonists may all result in selective receptor activation, individual ligands can preferentially activate a subset of the receptor’s downstream signalling pathways—a phenomenon known as biased agonism (Kise and Inoue, 2024). This is particularly relevant in cases where different endogenous hormones activate the same receptor (e.g., both CCK and gastrin activating CCK2R). Moreover, because synthetic analogues are often more stable and easier to synthesize than native peptide hormones, they are frequently used in studies investigating receptor function. However, these analogues may exhibit biased agonism, resulting in signalling outputs that differ from those elicited by the endogenous ligands. This may, in part, account for some of the divergent functional outcomes occasionally reported in the literature.

Moreover, biased agonists for various entero-pancreatic hormones are actively being developed, although their immune-modulating consequences remain largely unexplored. For example, ecnoglutide, a biased agonist for GLP-1R, has recently been shown to be both safe and effective in promoting weight loss (Ji et al., 2025). Ecnoglutide preferentially promotes cAMP elevation while avoiding β-arrestin recruitment. Since β-arrestin acts as a negative regulator of GPCRs such as GLP-1R, limiting its recruitment may enhance cAMP signalling intensity by reducing β-arrestin-mediated receptor desensitization. Similarly, CT-859, a dually biased GLP-1R/GIPR agonist, has been developed with the specific aim of curtailing β-arrestin recruitment (Rodriguez et al., 2025). Of note, however, β-arrestin has also been shown to physically interact with a variety of proteins beyond GPCRs, functioning as a scaffolding and adaptor protein in other signalling contexts (Kee et al., 2024). Importantly, β-arrestin has been implicated in a broad range of immune-related functions (Freedman and Shenoy, 2018). As these and other biased agonists continue to be developed, there is a clear need to better understand how such signalling pathways operate in immune cells, and how biased agonism may influence immune function.

## Immunoendocrinology beyond postprandial cAMP signalling

As discussed, postprandial endocrine response leads to an increase in entero-pancreatic hormones that reach the liver at elevated concentrations via the portal vein. This influx contributes to an anti-inflammatory and pro-tolerogenic immune environment in the liver, primarily through the elevation of intracellular cAMP levels. However, several of these hormones also activate signalling pathways independent of cAMP. In addition, the liver itself produces hepatokines whose expression is differentially regulated by feed-fast cycles and which may profoundly influence the hepatic immune environment through paracrine signalling. Glucagon and other hormones, such as ghrelin, are typically elevated during a fasted state. As discussed below, these endocrine factors may further contribute to the establishment of immune tolerance within the liver.

Insulin is a major postprandial hormone that not only facilitates the uptake of glucose and amino acids but also promotes an anabolic cellular state, driving the synthesis of macromolecules such as lipids and glycogen. In addition to its metabolic functions, insulin is a potent immune-modulating hormone (Van Niekerk et al., 2020). For instance, canonical insulin signalling through the PI3K/Akt/mTOR pathway is also activated by B-cell (Limon and Fruman, 2012) and T-cell (Tsai et al., 2018) receptors upon binding to recognized epitopes on MHC proteins. Insulin thus potentiates this immune-metabolic reprogramming necessary for immune function.

Furthermore, upon insulin binding, the insulin receptor can translocate to the nucleus, where it forms a complex with RNA polymerase II and other transcription factors to regulate the expression of various genes, including those involved in antiviral responses and epitope presentation (Hancock et al., 2019). These observations suggest a comprehensive role of insulin in promoting immune function. However, insulin receptors on lymphocytes are upregulated primarily following immune activation and are therefore not expected to exert strong immune-modulatory effects under steady-state conditions.

Postprandially, somatostatin is released by pancreatic δ cells as well as gastrointestinal D cells. Somatostatin suppresses the release of a variety of hormones and likely functions to inhibit both the secretion of fasting-associated hormones and to provide negative feedback on hormones released in response to feeding. There are five known somatostatin receptors (SSTR1–5), some of which can form heterodimers. However, the suppression of cAMP signalling appears to be the predominant function of SSTR activation (Zhang et al., 2025; Zhao et al., 2022). In addition to directly inhibiting cAMP, SSTRs also engage in crosstalk with other intracellular signalling pathways. For example, SSTR5 has been shown to modulate ERK1/2 and PI3K/Akt signalling (Somvanshi et al., 2011). It is therefore likely that the different receptor subtypes mediate distinct biological effects beyond the negative regulation of cAMP.

It has long been known that cells of both the innate and adaptive immune systems express different SSTR subtypes at varying levels (Lichtenauer-Kaligis et al., 2000). However, their specific roles in immune function remain poorly understood. In addition to receptor crosstalk, somatostatin also regulates the release of other hormones with established immune-regulatory functions. For example, SSTR2 is well studied, as agonists targeting this receptor are clinically used to suppress excessive growth hormone (GH) secretion in acromegaly, a condition characterized by abnormal growth of bones and organs. GH levels are typically elevated during prolonged fasting (e.g., overnight or during intermittent fasting) and have been shown to exert significant effects on immune function. In macrophages, for example, GH and IFNγ elicit overlapping transcriptional profiles, indicating that GH plays a pivotal role in supporting antimicrobial activity and antigen presentation (Schneider et al., 2019). Therefore, somatostatin, through its ability to suppress GH secretion, may represent an additional axis through which immune function is modulated.

Also of note, the liver is highly sensitive to GH stimulation, which drives the hepatic release of insulin-like growth factor 1 (IGF-1). IGF-1 has been shown to regulate immune function (Wang et al., 2024), adding another layer of complexity by suggesting that paracrine signalling via liver-derived hepatokines can shape the hepatic immune environment. Further complicating this regulatory network, IGF-1 and insulin receptors are capable of forming heterodimeric hybrid receptors that display a higher affinity for IGF-1 than for insulin (Slaaby, 2015).

The liver is also the primary source of circulating fibroblast growth factor 21 (FGF21), a fasting-induced hepatokine that has received considerable attention for its role in regulating metabolic adaptation to fasting. FGF21 promotes processes such as lipolysis and the expression of ketogenic genes, primarily through activation of the transcription factor PPARα (Reitman, 2007). More recently, FGF21 has also been shown to act in an autocrine/paracrine manner within the liver to promote lipophagy, thereby facilitating the mobilization of hepatic fatty acids (Byun et al., 2020). Notably, FGF21 has also been implicated in the regulation of immune function, suggesting that its local autocrine/paracrine effects in the liver may contribute to immune modulation.

For example, FGF21 has been reported to exert potent anti-inflammatory effects, suppressing the release of IL-1β and TNF following LPS challenge, while simultaneously enhancing the production of the anti-inflammatory cytokine IL-10 (Li et al., 2018). FGF21 knockout mice spontaneously develop inflammatory states in the lung and exhibit heightened sensitivity to LPS, a phenotype that can be ameliorated by administration of exogenous FGF21 (Cai et al., 2024). Furthermore, FGF21 has been shown to improve survival of liver grafts by modulating inflammation associated with ischemia-reperfusion injury (Yang et al., 2024). Indeed, most studies examining various pro-inflammatory conditions report an anti-inflammatory role for FGF21. However, some exceptions exist. For instance, in THP-1 monocyte/­macrophage cells, FGF21 promotes glucose uptake and ­activates the PI3K/Akt pathway (Wang et al., 2018). ­Similarly, enhanced phagocytosis and NADPH oxidase activity have been observed following FGF21 treatment (Wang et al., 2015). Thus, although the majority of ­evidence supports an anti-inflammatory function for FGF21, conflicting findings remain and warrant further investigation.

As previously noted, receptors for different receptor subtypes, or even different hormones, can occasionally form heterodimers, a phenomenon also reported to modulate cAMP signalling. For example, ghrelin, commonly referred to as the ‘hunger hormone’ due to its role in stimulating appetite and food intake, is secreted by X/A-like cells of the stomach. Although ghrelin is known to attenuate immune responses (Mathur et al., 2021), it is not typically associated with increased cAMP signalling. In fact, in pancreatic β cells, ghrelin has been shown to inhibit insulin secretion by suppressing cAMP levels (Kurashina et al., 2015). However, in striking contrast, co-expression of the ghrelin receptor (known as the growth hormone secretagogue receptor [GHS-R] due to ghrelin’s ability to stimulate GH release during fasting) with the dopamine 1 receptor (D1R) results in a shift in G-protein coupling: This heterodimerization leads to ghrelin enhancing dopamine signalling via increased cAMP accumulation (Jiang et al., 2006). This finding raises the possibility that immune cells co-expressing GHS-R and D1R may exhibit similarly enhanced cAMP responses. Notably, both T cells and other immune cell types have been shown to express D1R (Levite, 2016) as well as GHS-R (Dixit et al., 2004).

Here, we have focused primarily on entero-pancreatic hormones, particularly emphasizing the role of cAMP signalling. However, as the preceding discussion indicates, other hormones also contribute to shaping the immunological context of the liver. Insulin, for instance, is of particular interest as a potentially pro-inflammatory hormone. Other hormones, such as ghrelin, may acquire new functional relevance depending on the cellular context, particularly in relation to receptor expression and receptor heterodimerization. GH, although not delivered directly to the liver via the portal vein, may nonetheless influence hepatic immune function through systemic actions or local indirect mechanisms, for example, via paracrine signalling from IGF-1 acting on liver-resident immune cells. FGF21 is also likely to exert significant effects on the liver’s immunological environment through paracrine activity. Although initial interest in FGF21 focused on its metabolic roles, it is now evident that this hepatokine has substantial immunomodulatory effects, even if the underlying mechanisms remain incompletely understood.

Finally, another interesting question is how fasting and feeding cycles differentially affect liver tolerance. During fasting, anti-inflammatory endocrine factors such as glucagon and FGF21 are likely to further promote liver tolerance in the absence of cAMP-inducing hormones. Ghrelin, another gastrointestinal hormone released during fasting, has also been shown to exert anti-inflammatory effects, while simultaneously stimulating the release of GH, which appears to be more pro-inflammatory. How these and other hormones differentially impact liver function during feeding-fasting cycles remains to be explored.

## Implications

**Figure 4.**
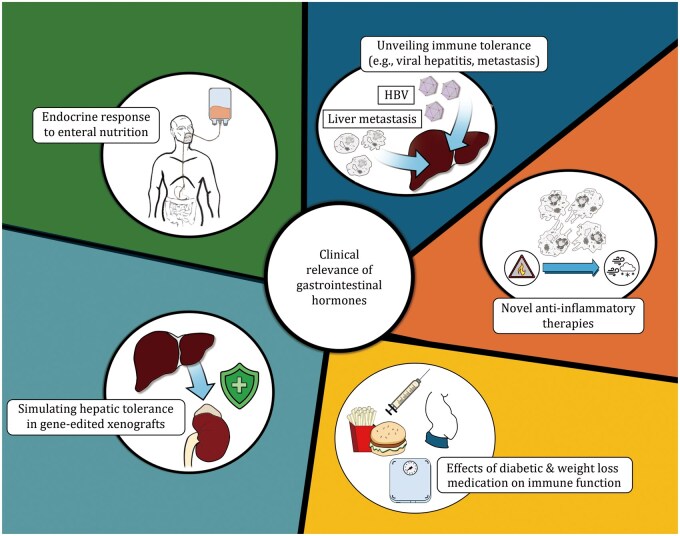
A better understanding of how endocrine factors released following a meal impact on immune function may have implications in a number of clinical settings.

Taken together, it is clear that elucidating the immune-modulating effects of these endocrine factors could have far-reaching implications (Fig. 4). A number of entero-pancreatic peptides, such as GLP-1, glucagon, GIP, and amylin, which activate Gαs-coupled GPCRs (i.e., signal via cAMP), are targeted in current GLP-1-based multi-agonist therapies for diabetes and as weight-loss agents. Notably, however, these agents have also demonstrated therapeutic utility beyond weight management and diabetes (reviewed in Drucker, 2025). For example, Retatrutide (GLP-1/GIP/glucagon) has been shown to effectively reduce liver fat content in metabolic dysfunction-associated steatotic liver disease (MASLD) (Sanyal et al., 2024). Tirzepatide (GLP-1/GIP), initially developed for weight loss, is now also being investigated for chronic kidney disease (Packer et al., 2025) and metabolic dysfunction-associated steatohepatitis (Loomba et al., 2024). Similarly, this class of hormone receptor agonists has also been shown to confer benefits in atherosclerosis and cardiovascular disease (reviewed in Marx et al., 2022).

Part of these beneficial effects can be directly attributed to weight loss and reduced food intake (e.g., lower cholesterol and sugar consumption). However, because inflammation is a common co-occurring feature in many of these pathological states, it is increasingly recognized that the anti-inflammatory properties of this class of hormone-based drugs may play a significant role in their efficacy across such a broad range of clinical settings (Drucker, 2024; Marx et al., 2022). For example, studies in transgenic mouse models have demonstrated that the beneficial effects of GLP-1 agonists such as semaglutide on plaque lesion formation are at least partly mediated independently of weight reduction and cholesterol-lowering effects (Rakipovski et al., 2018). This work highlighted that a reduced inflammatory tone resulted in altered leukocyte behaviours central to the pathogenesis of atherosclerosis (e.g., leukocyte rolling, extravasation, and cholesterol metabolism), which played a key role in mediating the anti-atherosclerotic effects of semaglutide (Rakipovski et al., 2018). Similarly, we have suggested that the recently reported effectiveness of pemvidutide, a GLP-1/glucagon dual receptor agonist, in treating MASLD may in part relate to its anti-inflammatory effects (van Niekerk et al., 2025). For instance, inflammation is a key driver of *de novo* lipogenesis in the liver, suggesting a synergistic benefit of pemvidutide in attenuating hepatic lipid accumulation. Thus, although these drugs likely act through multiple parallel mechanisms, their anti-inflammatory effects warrant particular attention.

These observations not only reinforce the anti-inflammatory potential of entero-pancreatic peptide hormones but also indicate that this class of drugs could be repurposed as immune-modulating therapies. However, the chronic administration of these receptor agonists at supraphysiological doses raises important immunological questions: What effects might such treatments have on susceptibility to infection or on the efficacy of vaccine responses? Moreover, advances in drug delivery, such as drug-loaded hydrogels, may further reduce the frequency of administration from weekly injections to once every few months. This means that individuals undergoing unscheduled medical interventions (e.g., developing sepsis) may have these agents active in their system. These considerations highlight the need to understanding how these drugs might affect immune function.

Another intriguing possibility lies in the development of novel strategies for engineering xenografts, a field that remains challenging despite incremental progress (Griffith et al., 2025). Constitutive expression of gastrointestinal peptide hormones in transplanted organs might offer a means to create ‘immunologically veiled’ xenografts by establishing a paracrine environment that promotes local immune tolerance. Conversely, disrupting the tolerogenic environment of the liver could provide new approaches for treating liver infections (viral hepatitis), or even metastatic lesions.

A deeper understanding of appetite suppression during infection may also shed light on a longstanding question: Why do we lose our appetite when we fall ill? Appetite loss is a well-recognized component of sickness behaviour and is commonly observed during infection. It is tempting to speculate that this response serves to circumvent the immune-modulatory effects of the endocrine response to feeding. In this context, nutrient intake, particularly enteral nutrition during infection, such as in cases of sepsis, may elicit a postprandial endocrine response that could potentially interfere with optimal immune function. Given the ongoing debate surrounding nutritional support in sepsis, these considerations suggest that, in certain circumstances, permissive underfeeding may confer therapeutic benefits (van Niekerk et al., 2020).

The liver, as the largest internal organ, contains approximately 10%–15% of the total blood volume. Owing to its low-pressure, percolative blood flow, circulating immune cells entering the liver have ample opportunity to interact with hepatic cells. In particular, LSECs play a key role in recruiting and ‘trapping’ immune cells within the hepatic microvasculature (Shetty et al., 2018). Consequently, the liver may play an important role in modulating systemic inflammation by reprogramming immune cells that transit through this tolerogenic compartment. The observation that liver-targeted mannosylation of allergen-derived antigens induces Tregs that attenuate immune activation in the respiratory tract (Emiliano Gómez Medellín et al., 2025) further underscores that the tolerogenic compartment of the liver can have systemic consequences.

Critically, it should be emphasized that, although it has long been recognized that immune cells express receptors for these endocrine factors, their role in immune function remains poorly characterized. Furthermore, some of the available studies are dated and rely on methodologies that have since been superseded by more advanced techniques. In addition, the functions of these endocrine factors within the liver, and their effects on various hepatic cell types (e.g., hepatocytes, Kupffer cells, LSECs, liver-resident NK cells, etc.) remain largely unexplored. Given the potential consequences of such tolerogenic systems, there is a clear and pressing need to develop a more comprehensive understanding of how these entero-pancreatic endocrine factors regulate immune function.

Because of their involvement in other physiological processes, there are likely to be situations in which the therapeutic application of these agents may be limited. Numerous clinical trials have demonstrated that several peptide hormones, including glucagon, amylin, GIP, and GLP-1, are well tolerated and possess favourable pharmacological profiles. However, since the use of these agents suppresses appetite to the extent that muscle loss can also occur (Prado et al., 2024), their immune-regulatory utility may be restricted. For example, promoting placental tolerance or treating inflammation in cachexia would require careful monitoring of food intake.

Other peptide hormones, such as CCK, have also been explored for weight loss, though with less success than GLP-1. This is likely because CCK is more effective at inducing meal termination (acute satiation) than at suppressing long-term food intake and thereby facilitating weight loss (Warrilow et al., 2023). Moreover, due to CCK’s role in the central nervous system, analogues may induce side effects such as anxiety (Ballaz et al., 2020), while its gastric actions can lead to excessive pancreatic and biliary secretion, resulting in diarrhoea and electrolyte disturbances. Similarly, based on the clinical features of VIPoma syndrome (VIP-secreting tumours) (Abdullayeva, 2019), it is likely that chronically elevated VIP could cause watery diarrhoea and electrolyte imbalances, accompanied by vascular complications such as hypotension and flushing. Thus, it remains uncertain whether the desired immune-modulating effects of such peptides can be achieved at the dose and duration required for effective therapeutic application.

Similar caution is warranted for BAs and their analogues. For example, in the livers of mice, activation of the BA receptor FXR transcriptionally suppresses autophagy, even under fasting conditions (Seok et al., 2014). This BA-mediated suppression via FXR likely reflects the requirement for the liver to shift into an anabolic state to consolidate incoming nutrients, a process for which catabolic pathways such as autophagy may be counterproductive. However, evidence suggests that impaired autophagy predisposes to metabolic-associated liver pathology (Baselli et al., 2022; Ren et al., 2024). For instance, lipophagy has been shown to facilitate the clearance of lipids from the liver (Minami et al., 2023). In addition, the autophagy–lysosomal pathway appears to play an important role in mediating LPS tolerance in the liver, since lysosomal enzymes such as acyloxyacyl hydrolase are essential for LPS detoxification (Liu et al., 2020). Taken together, these observations highlight a potential mechanism by which BAs, through FXR-mediated suppression of autophagy, may enhance LPS sensitivity and thereby increase inflammatory tone.

## Abbreviations

AKAPs, A-kinase anchoring proteins; AC, adenylyl cyclase; BAs, bile acids; cAMP, cyclic AMP; CRE, cAMP response ­elements; CREM, cAMP-responsive element modulator; CCK, cholecystokinin; DAMPs, damage-associated molecular patterns; DCs, dendritic cells; D1R, dopamine 1 receptor; Epac, exchange protein directly activated by cAMP; FXR, farnesoid X receptor; FGF21, fibroblast growth factor 21; GPCRs, G protein-coupled receptors; GLP-1, glucagon-like peptide-1; GLP-1RAs, GLP-1 receptor agonists; GIP, glucose-dependent insulinotropic polypeptide; GIPR, GIP receptor; GH, growth hormone; GHS-R, growth hormone secretagogue receptor; GEFs, guanine nucleotide exchange factors; ICER, inducible cAMP early repressor; IGF-1, insulin-like growth factor 1; LSECs, liver sinusoidal endothelial cells; mTORC1, mammalian target of rapamycin complex 1; PXR, pregnane X receptor; PGE2, prostaglandin E2; PKA, protein kinase A; RA, retinoic acid; RORγ, retinoic acid receptor-related orphan receptor γ; SSTR, somatostatin receptor; TGR5, Takeda G protein-coupled receptor 5, VIP, vasoactive intestinal peptid
